# Impact of three variants of prolonged exposure therapy on comorbid diagnoses in patients with childhood abuse-related PTSD

**DOI:** 10.1080/16506073.2024.2318729

**Published:** 2024-02-27

**Authors:** Chris M. Hoeboer, Marie-Louise J. Kullberg, Danielle A.C. Oprel, Maartje Schoorl, Agnes van Minnen, Niki Antypa, Joanne Mouthaan, Rianne A. de Kleine, Willem van der Does

**Affiliations:** a Department of Clinical Psychology, Leiden University, Leiden, The Netherlands; b Department of Psychiatry, Amsterdam UMC location University of Amsterdam, Amsterdam, The Netherlands; c PsyQ, Parnassia Groep, The Hague, The Netherlands; d PSYTREC, Bilthoven, The Netherlands; e Behavioural Science Institute, Radboud University, Nijmegen, The Netherlands

**Keywords:** Prolonged exposure, childhood-abuse related PTSD, posttraumatic stress disorder, comorbid diagnoses, depression, anxiety

## Abstract

Recent studies indicated that Prolonged Exposure (PE) is safe and effective for posttraumatic stress disorder (PTSD). It is unclear whether PE also leads to a reduction in comorbid diagnoses. Data from a large randomized controlled trial (*N* = 149) on the effects of three variants of PE for PTSD were used. We examined the treatment effects on co-morbid diagnoses of depressive, anxiety, obsessive compulsive, substance abuse, psychotic, eating and personality disorders in a sample of patients with PTSD related to childhood abuse. Outcomes were assessed with clinical interviews at baseline, post-treatment and at 6- and 12-month follow-up. All variants of PE led to a decrease from baseline to post-treatment in diagnoses of depressive, anxiety, substance use and personality disorders. Improvements were sustained during follow-up. We found an additional decrease in the number of patients that fulfilled the diagnostic criteria of a depressive disorder between 6- and 12-month follow-up. No significant changes were observed for the presence of OCD, psychotic and eating disorders. Findings suggest that it is effective to treat PTSD related to childhood abuse with trauma-focused treatments since our 14-to-16 weeks PE for PTSD resulted in reductions in comorbid diagnoses of depressive, anxiety, substance use and personality disorders.

## Background

Prolonged exposure (PE) is an effective and safe trauma-focused treatment (TFT) of posttraumatic stress disorder (PTSD; Martin et al., 2021; Mavranezouli et al., 2020; Oprel et al., 2021; Watkins et al., 2018) and listed as one of the preferred interventions in treatment guidelines (Card, 2017; Hamblen et al., 2019). PE is a form of cognitive behavioral therapy during which patients process the traumatic memories and learn that memories and trauma-related cues are safe and that they can cope with them. PE involves psychoeducation about PTSD, imaginal exposure (repeatedly recounting the most disturbing traumatic memories) and exposure in vivo (repeatedly approaching trauma-related stimuli; Foa et al., 2007). Despite its established safety and effectiveness, PE is still greatly underutilized (Borah et al., 2013; McLean & Foa, 2013; van Minnen et al., 2010). One important reason is that clinicians are often reluctant to use PE in patients with PTSD when they also meet criteria for comorbid diagnoses. The main concern is that the comorbid psychopathology may exacerbate (Becker et al., 2004; Trottier et al., 2017; van Minnen et al., 2010). Empirical evidence, however, points towards the opposite. That is, a reduction in comorbid symptomatology alongside PTSD symptoms often occurs during TFT (van Minnen et al., 2015). Because studies on the effect of TFT on comorbid disorders thus far reported mainly on changes in self-reported symptom severity of comorbid conditions, still little is known about changes in comorbid diagnostic status. This information is needed to inform clinicians and researchers about the use of PE for PTSD with comorbid conditions. The current study therefore focused on the effects of PE on the most prevalent comorbid disorders in this patient group, namely depressive disorders, anxiety disorders, obsessive compulsive disorder (OCD), substance abuse disorders, psychotic disorders, eating disorders and personality disorders.

### Prevalence and treatment of comorbid disorders in patients with PTSD

#### Depressive disorders

With a lifetime prevalence rate of 49%, comorbidity between PTSD and depressive disorders is high (Walter et al., 2018). Specifically, between 48% and 55% of the persons with PTSD meet criteria for a comorbid Major Depressive Disorder (MDD) and 21% to 23% meet criteria or dysthymia (Kessler et al., 1995). In previous studies, trauma-focused behavioral therapy in patients with PTSD and comorbid depressive symptoms resulted in reductions in self-reported depressive symptoms along the decline of PTSD symptoms (Knutsen et al., 2018; Larsen et al., 2019). During PE, changes in PTSD symptoms led to changes in depressive symptoms to a greater extent than vice versa (Aderka et al., 2013; McLean et al., 2017). There are no studies published on the effect of any form of TFT on loss of diagnoses of depressive disorders.

#### Anxiety disorders

About 59% of the patients with PTSD meet criteria for one or more anxiety disorders (i.e. panic disorder, social anxiety disorder, generalized anxiety disorder, specific phobia) (Pietrzak et al., 2011). More specifically, prevalence rates of different types of anxiety disorders range between 19.4% and 37.3% (Pietrzak et al., 2011). Studies on PE and TF-CBT in patients with PTSD and comorbid anxiety disorders reported a reduction in self-reported general anxiety symptoms after treatment (Larsen et al., 2019; van Minnen et al., 2015). There are no studies published on the effect of TFT on the loss of diagnoses of anxiety disorders.

#### Obsessive-compulsive disorder (OCD)

Studies report prevalence rates ranging from 30% to 41% (Badour et al., 2012; Gershuny et al., 2008; Nacasch et al., 2011). The prevalence of OCD in patients with PTSD remains relatively understudied in the current literature, and no meta-analytic evidence or evidence from large-scale national epidemiologic studies is available yet. The effects of TFT on symptoms or diagnosis of OCD have, to the best of our knowledge, not been investigated yet.

#### Substance use disorders (SUDs)

Comorbidity rates of substance use disorders in PTSD are high (up to 49%; Breslau et al., 2003; Kessler et al., 1995; Pietrzak et al., 2011; Walter et al., 2018). Among persons with PTSD, the most frequently used substances were alcohol, sedative drugs, and cannabis (Kessler et al., 1995; Walter et al., 2018). A recent meta-analysis summarized the effectiveness of treatment targeting both PTSD and SUDs such as Concurrent Treatment of PTSD and Substance Use Disorders Using Prolonged Exposure (COPE; Norman et al., 2019). They found that TFT, non TFT, and manualized SUD interventions were effective in targeting SUD and PTSD (Simpson et al., 2021). TFT outperformed other treatment options on PTSD outcomes post-treatment, but manualized SUD interventions resulted in a larger reduction in substance use compared to TFTs. Little research has explored whether TFT, targeting only PTSD symptoms, is effective in reducing SUD. However, it has been demonstrated that improvements in PTSD symptomatology are associated with subsequent declines in substance use (Back et al., 2006; Hien et al., 2010).

#### Psychotic disorders

Among patients with PTSD, psychotic symptomatology, such as auditory and visual hallucinations and delusions, frequently occurs with great variability between studies (15% to 64%; Braakman et al., 2009). The presence of a full-blown psychotic disorder in persons with PTSD was 2.8% (Walter et al., 2018). In a meta-analysis of 12 studies, including patients with comorbid psychotic disorders and PTSD, TFT reduced positive symptoms immediately after treatment, but this effect was not maintained at follow-up (Brand et al., 2018). In the same study, it was found that TFT also reduced delusions, but no significant effects on hallucinations and negative symptoms were found. Moreover, it has been demonstrated that, as a result of PE and Eye Movement Desensitization and Reprocessing (EMDR), more patients achieved remission of their schizophrenia as compared to the waiting list control condition (de Bont et al., 2016), and this effect was maintained at one-year follow-up (van den Berg et al., 2018).

#### Eating disorders

Findings from a recent quantitative synthesis showed that the pooled prevalence of the eating disorders (EDs) anorexia nervosa and bulimia nervosa among patients with PTSD was 10.4% and 22.4%, respectively (Ferrel et al., 2022). It has been argued that treatment needs to address both PTSD and ED to successfully recover from both disorders (Brewerton, 2007). Recently, an RCT was published which was the first study addressing the effects of cognitive behavioral therapy (CBT) on PTSD in patients with an eating disorder after intensive eating disorder treatment (Trottier et al., 2022). It was found that CBT was associated with improvements in PTSD. However, the study was not powered to detect changes in diagnoses of eating disorders.

#### Personality disorders

Meta-analytic evidence revealed that 35% of the persons with PTSD had at least one personality disorder (Friborg et al., 2013). The most common diagnoses were paranoid (26%), avoidant (23%), and borderline personality disorders (22%). Meta-analytic evidence shows that patients with PTSD and a comorbid personality disorder were not at higher risk for dropout from TFT; however, they might benefit less from TFT compared to patients without a comorbid personality disorder (Snoek et al., 2021). In studies using EMDR and Narrative Exposure Therapy (NET) in patients with personality disorders, it was found that borderline personality disorder symptoms and psychological distress declined and personality functioning improved during treatment (De Jongh et al., 2020; Steuwe et al., 2016). Recent findings show that a combination of PE with EMDR resulted in a decrease of comorbid borderline personality disorder diagnoses at the 12-month follow-up (Kolthof et al., 2022). However, findings on the effects of PE only and in patients with PTSD with other personality disorders are lacking.

It should be noted that most studies investigating the effect of PE on comorbid conditions measured self-reported symptomatology rather than clinician-assessed disorders. One reason that studies on changes in co-occurring clinician-assessed diagnoses (as opposed to self-reported symptoms) are lacking is that binary outcomes often require more power than continuous outcomes and most RCTs are powered for continuous outcomes. However, using self-reported symptomatology in such studies has several limitations, including social desirability and recall bias (together often referred to as *self-reporting bias*) precluding valid and reliable conclusions about the treatment effect on psychopathology (Althubaiti, 2016). Even more importantly, it is difficult or even impossible for patients to differentiate between symptoms from distinct disorders when reporting using self-report measures, which might contribute to the high comorbidity rates between PTSD and conditions such as anxiety disorders, depression and OCD (Engelhard et al., 2007; Franklin & Raines, 2019). Therefore, when only measuring symptoms via self-report measures, a decrease in comorbid conditions might simply reflect the decrease in PTSD symptoms rather than an effect of treatment beyond PTSD.

### Present study

Despite the high prevalence of co-occurring conditions among patients with PTSD, changes in the diagnostic status of these comorbid disorders after PTSD treatment using clinical interviews have rarely been assessed. In the present study, we aim to investigate the effects of PE on clinician-assessed comorbid psychopathology in patients with childhood abuse-related posttraumatic stress disorder (CA-PTSD). Specifically, we will examine change in diagnoses of current depressive disorders, anxiety disorders, personality disorders, OCD, eating disorders, psychotic disorders and SUDs as examined with established clinical diagnostic interviews, the Mini-International Neuropsychiatric Interview (MINI; Sheehan et al., 1998) and the Structured Clinical Interview for DSM-IV (SCID-2; Weertman et al., 2003) We will also explore the change in specific disorders (e.g. agoraphobia or borderline personality disorder) when at least 20 patients met criteria for the disorder at baseline. This study contains data collected from a randomized-controlled trial (Oprel et al., 2018) comparing the effectiveness of three variants of exposure-based therapy: PE (Foa et al., 2007), intensified PE (iPE) and PE preceded by Skills Training in Affective and Interpersonal Regulation (STAIR+PE; Cloitre et al., 2002; Levitt & Cloitre, 2005) in patients with PTSD related to childhood abuse. The treatment effect, i.e. PE, iPE and STAIR+PE, on the primary outcome (PTSD symptom severity) is large (Oprel et al., 2021).

## Methods

### Study design and procedures

One hundred forty-nine patients participated in the RCT that compared the effectiveness of PE, i-PE and STAIR+PE. Detailed information on the study design and procedures of the IMPACT-study can be found in the published study protocol (Oprel et al., 2018) and the published article on the main outcomes of the RCT (Oprel et al., 2021). Participants were recruited from two outpatient mental health services specializing in the treatment of trauma-related disorders located in The Hague and Rotterdam, the Netherlands. Inclusion criteria were: 1) ages 18–65 years; 2) a PTSD diagnosis according to the DSM-5 classification established with the Clinician-Administered PTSD Scale (CAPS-5 see below), and at least moderate severity of PTSD-symptoms (CAPS-5 score ≥ 26) and at least one specific memory of the traumatic event; 3) traumata related to childhood sexual and/or physical abuse that occurred before 18 years of age, committed by a primary caretaker or an authority figure as index event; 4) sufficient fluency in Dutch to complete the treatment and research protocols. Exclusion criteria were: 1) involvement in a compensation case or legal procedures concerning admission or stay in The Netherlands; 2) pregnancy (to ensure the treatment could be delivered in full); 3) severe nonsuicidal self-injury (NSSI) which required hospitalization during the past 3 months; 4) severe suicidal behavior: a suicide attempt during the past 3 months or acute suicidal ideations with serious intent to die with a specific plan for suicide and preparatory acts; 5) severe disorder in the use of alcohol or drugs in last 3 months according to the MINI; 6) cognitive impairment (estimated IQ < 70); 7) changes in psychotropic medication in the 2 months prior to inclusion; and 8) engagement in any current psychological treatment. Written informed consent was obtained from all patients after they received a complete description of the study. In this study, PE consisted of weekly face-to-face sessions of 90 minutes. During the sessions, patients receive imaginal exposure (repeatedly and systematically recounting the most distressing traumatic memories) and exposure in vivo (repeatedly approaching trauma-related stimuli). iPE started with three sessions per week for 4 weeks (12 sessions total) followed by two sessions (one after 1 month and one after 2 months). Apart from the format, the session content was the same as in the PE condition. STAIR+PE consisted of eight weekly face-to-face sessions of 60 minutes for STAIR and eight weekly face-to-face sessions of 90 minutes for PE. The first half of the treatment (STAIR) involves a skills training with four sessions focused on emotion regulation and four sessions focused on interpersonal skills. The second half of the treatment includes PE as in the PE and iPE conditions, but fewer sessions. Between November 2016 and December 2018, 150 participants were randomly assigned to PE, iPE or STAIR+PE. One participant was excluded after randomization because they no longer met inclusion criteria at the time of enrolment. In total, 37 patients (25%) dropped out of treatment. We found no demographic or clinical characteristics which were related to dropout from therapy. Little’s MCAR test indicates that missing cases may meet criteria for missing completely at random (*χ*^*2*^(244) = 241, *p* = .54). More detailed information about the design can be found in the published study protocol (Oprel et al., 2018). The authors assert that all procedures contributing to this work complied with the ethical standards of the relevant national and institutional committees on human experimentation and with the Helsinki Declaration of 1975, as revised in 2008. All procedures involving patients were approved by the Medical Ethical Committee of Leiden University Medical Centre (NL57984.058.16).

### Sample

At baseline (T0), participants’ age ranged between 20 and 60 years, with a mean age of 36.9 (*SD* = 11.8) and 76.5% of the sample was female. Of the total sample, 20.1% finished higher vocational education or university and 43.3% had a non-Western cultural background (i.e. has at least one parent who was not born in a western country). With regard to trauma history, 72.5% of the participants reported childhood sexual abuse, 62.4% reported childhood physical abuse, 19.5% reported sexual abuse in adulthood and 28.2% reported physical abuse in adulthood. The mean duration of PTSD was 15.1 (*SD* = 12.5) years. About half of the patients (80; 54%) met criteria for complex PTSD according to the International Trauma Questionnaire (ITQ; Cloitre et al., 2018). See also Hoeboer et al. (2021) for more details on the effect of Complex PTSD on treatment outcomes in this study.

### Measures

#### Mini-International Neuropsychiatric Interview (MINI)

Comorbid axis-1 disorders according to the DSM-IV were assessed with the MINI (Sheehan et al., 1998) at T0 (baseline), T3 (after 16 weeks/post-treatment), T4 (at 6-month follow-up) and T5 (at 12-month follow-up). The MINI is a structured interview with closed-end questions based on DSM-IV and ICD-10. Its inter-rater reliability proved to be good (kappa values of all diagnostic subscales are above .75; Sheehan et al., 1998).

#### Structured Clinical Interview for DSM-IV Personality Disorders (SCID-2)

Personality disorders were assessed with the Structured Clinical Interview for DSM-IV Personality Disorders (SCID-2; Weertman et al., 2003) at T0 (baseline) and T4 (at 6-month follow-up). The reliability of the Dutch version of the SCID-2 was shown to be good: in an outpatient population, kappas ranged from .77 for obsessive—compulsive personality disorder to .82 for avoidant personality disorder. Weighted kappa for all personality disorders was .80. The inter-rater agreement proved to be fair to excellent (Intraclass Correlation Coefficients (ICC) ranging from .41 to .88), except for the dependent personality disorder (ICC < .40; Weertman et al., 2003).

All interviews were administered by trained research assistants who received weekly supervision.

### Power calculation

Power simulations were performed using the R package “simr” to assess the probability of detecting an effect when this effect is truly present (note that this power calculation was carried out post-hoc since the original sample size was based on power calculations for the main outcome of the trial, i.e. PTSD symptom reduction). Given 100 simulations with a data frame of 150 participants with four measurements, 25% random missing assessments, an unbalanced outcome where 25% of the participants meet criteria of a disorder at baseline, an intraclass correlation of .33 (corresponding to moderate variance being explained by the random effect), residual variance of 1.4 (referring to the within-person variance which accounts for 67% of the total variance) and a decrease of the outcome with a small-medium effect size (odds ratio of .50 corresponding to 50% decrease in the occurrence of a disorder after treatment) from the baseline to first assessment followed by no change in the remaining assessments, power was estimated to be 99% using a threshold for significance of *p* < .05; 95% using a threshold for significance of *p* < .0135 (used to adjust for multiple testing) and 86% using a threshold of *p* < .009 (used to adjust for multiple testing in the subgroup analyses).

### Statistical analyses

Comorbid axis-1 disorders were assessed at baseline (T0), post-treatment after 16 weeks (T3) and at a 6- and 12-month follow-up (T4 and T5 respectively). Personality disorders were only assessed at T0 and T4. Prior to the analysis, a statistical plan was preregistered at the Open Science Framework (https://osf.io/uhcdp/). All analyses were carried out in R version 4.2.1 (R Core Team, 2019) with the lme4-package (Bates et al., 2014). Analyses were based on an intention-to-treat basis. Missing data was taken into account using maximum likelihood estimation. For all analyses, alpha was set to .05 (two-sided) and the treatment condition was dummy coded with PE as comparator condition. We corrected for multiple testing using the Benjamini Hochberg method (Benjamini, 2010).

For all analyses, generalized linear mixed effect models were applied to account for repeated measures and estimate treatment effects on comorbid diagnoses over time. All outcomes except for personality disorders are estimated using a piecewise growth curve model with the first slope indicating the treatment effect (T0 to T3), the second slope indicating the first follow-up trajectory (T3 to T4) and the third slope indicating the second follow-up trajectory (T4 tot T5) to account for non-linearity. Models for personality disorders are estimated with a generalized linear mixed-effect model with one slope indicating the treatment effect (T0 to T4). All models include random intercepts. The comorbid condition (presence of current depressive disorders, anxiety disorders, personality disorders, OCD, eating disorders, psychotic disorders and SUDs) was the dependent variable in the analysis with condition and the two slopes added as independent variables. As preregistered at OSF, in the explorative subgroup specificity analyses, we used specific disorders (e.g. agoraphobia or borderline personality disorder) as outcomes when at least 20 patients met criteria for the disorder at baseline. We also performed a sensitivity analysis repeating all previous models with the interaction effects between the two slopes and treatment conditions as additional independent variables to check whether the change in comorbid conditions differed between conditions.

## Results

The presence of comorbid psychiatric disorders at baseline and post-treatment is described in Table 1.

**Table 1.** Comorbid psychiatric disorders at baseline (*N* = 149) and post-treatment (*N* = 96).

		Baseline	Post-treatment
		% (N)	% (N)
Any depressive disorder		65.8 (98)	51.5 (53)
	MDD	57.0 (85)	35.0 (36)
	Dysthymia	8.7 (13)	18.4 (19)
Any anxiety disorder		77.2 (115)	52.4 (54)
	Agoraphobia	53.7 (80)	30.1 (31)
	Social anxiety disorder	38.3 (57)	27.2 (28)
	Panic disorder	36.9 (55)	17.5 (18)
	GAD	25.5 (38)	14.6 (15)
	Simple phobia	16.8 (25)	11.7 (12)
OCD		24.8 (37)	13.6 (14)
Psychotic disorder		12.8 (19)	7.8 (8)
SUD		22.8 (34)	8.7 (9)
	Alcohol use disorder	14.8 (22)	3.8 (4)
	Drug use disorder	14.1 (21)	5.8 (6)
Eating disorder		6.0 (9)	7.8 (8)
	Anorexia nervosa	0.0 (0)	0.0 (0)
	Bulimia	6.0 (9)	7.8 (8)
Any personality disorder		61.1 (91)	41.6 (42)
	Avoidant personality disorder	34.2 (51)	19.8 (20)
	Borderline personality disorder	30.8 (31)	16.8 (17)
	Paranoid personality disorder	19.5 (29)	8.9 (9)
	Obsessive compulsive personality disorder	13.4 (20)	10.8 (11)
	Personality disorder NOS	9.4 (14)	5.9 (6)
	Antisocial personality disorder	8.1 (12)	4.0 (4)
	Dependent personality disorder	6.7 (10)	5.0 (5)
	Schizotypical personality disorder	5.4 (8)	4.0 (4)
	Schizoid personality disorder	3.4 (5)	4.0 (4)
	Narcissitic personality disorder	1.3 (2)	0.0 (0)
	Histrionic personality disorder	0.7 (1)	1.0 (0)

MDD = major depressive disorder, GAD = generalized anxiety disorder, OCD, SUD, NOS = not otherwise specified, note that post-treatment is T3 for outcomes assessed with the MINI and T4 for outcomes assessed with the SCID-2.

### Effects of PE on comorbid disorders

We found that fewer participants met criteria for depressive, anxiety, substance use and personality disorders between baseline and post-treatment (all p-values < .01). The number of participants with a depressive disorder also decreased between 6- and 12-month follow-up (*p* = 0.013). Thus, at post-treatment, fewer participants met the criteria for these disorders compared to the baseline. No changes in the number of participants with OCD, psychotic disorders or eating disorders were found. Table 2 shows the results of the generalized linear mixed effect model analyses with bootstrapped 95% confidence intervals and effect sizes. We did not find that changes in any of the comorbid diagnoses were larger in one treatment condition compared to the others.

**Table 2.** Main outcomes of the generalized mixed linear models.

Model	Disorder	Slope	Estimate	Standard error	p	q	OR [95% CI]
1	Depressive disorders	T0-T3	−0.87	0.34	0.010	0.011*	0.42 [0.21, 0.81]
		T3-T4	−0.34	0.35	0.332	0.034	0.71 [0.36, 1.42]
		T4-T5	−0.94	0.38	0.013	0.013*	0.39 [0.19, 0.82]
2	Anxiety disorders	T0-T3	−1.71	0.38	<.001	0.003*	0.18 [0.09, 0.38]
		T3-T4	−0.40	0.37	0.276	0.029	0.67 [0.33, 1.37]
		T4-T5	−0.20	0.37	0.584	0.042	0.82 [0.39, 1.69]
3	OCD	T0-T3	−0.95	0.40	0.016	0.016	0.39 [0.18, 0.84]
		T3-T4	−0.15	0.46	0.746	0.050	0.86 [0.35, 2.14]
		T4-T5	0.39	0.46	0.405	0.037	1.47 [0.59, 3.63]
4	SUDs	T0-T3	−2.62	0.84	0.002	0.008*	0.07 [0.01, 0.38]
		T3-T4	0.89	0.82	0.278	0.032	2.43 [0.49, 12.10]
		T4-T5	−1.09	0.85	0.200	0.024	0.34 [0.06, 1.78]
5	Psychotic disorders	T0-T3	−0.92	0.71	0.195	0.021	0.40 [0.10, 1.60]
		T3-T4	0.40	0.75	0.596	0.045	1.49 [0.34, 6.53]
		T4-T5	−1.77	0.94	0.060	0.018	0.17 [0.03, 1.08]
6	Eating disorders	T0-T3	0.39	0.90	0.660	0.047	1.48 [0.26, 8.55]
		T3-T4	−0.76	0.98	0.438	0.039	0.47 [0.07, 3.18]
		T4-T5	−1.38	1.21	0.254	0.026	0.25 [0.02, 2.69]
7	Any personality disorder	T0-T4	−1.21	0.38	0.002	0.005*	0.30 [0.14, 0.63]

*= Significant effect, Benjamini Hochberg corrected significance level q < .0135, OR = odds ratio, 95% CI = 95% confidence interval, OCD = obsessive-compulsive disorder, SUDs = substance use disorders.

In Table 3, the results of subgroup analyses for comorbid disorders with at least 20 participants meeting the criteria at baseline are presented. We found that MDD, agoraphobia, panic disorder, alcohol use disorder and avoidant personality disorder decreased from baseline to post-treatment (all *p*-values < .01). No significant changes in the presence of social anxiety disorder, drug use disorder, paranoid personality disorder, obsessive-compulsive personality disorder and borderline personality disorder diagnoses were found. Also no changes were found for any of the disorders during the follow-up period.

**Table 3.** Specificity analyses of the generalized mixed linear models (subgroups *n* > 20).

Model	Disorder	Slope	Estimate	Standard error	p	q	OR [95% CI]
1	MDD	T0–3	1.33	0.35	<.001	0.002*	0.26 [0.13, 0.52]
		T3–4	−0.23	0.37	0.530	0.420	0.80 [0.39, 1.63]
		T4–5	−0.43	0.39	0.268	0.030	0.65 [0.30, 1.39]
2	Agoraphobia	T0–3	−1.48	0.37	<.001	0.005*	0.23 [0.11, 0.46]
		T3–4	−0.30	0.39	0.452	0.036	0.74 [0.35, 1.61]
		T4–5	−0.59	0.43	0.169	0.024	0.55 [0.24, 1.28]
3	Social anxiety disorder	T0–3	−0.86	0.36	0.017	0.011	0.42 [0.21, 0.86]
		T3–4	−0.46	0.40	0.250	0.028	0.63 [0.29, 1.38]
		T4–5	0.28	0.41	0.495	0.038	1.32 [0.59, 2.95]
4	Panic disorder	T0–3	−1.49	0.42	<.001	0.004*	0.23 [0.10, 0.51]
		T3–4	0.24	0.45	0.596	0.044	1.27 [0.52, 3.07]
		T4–5	−0.63	0.48	0.194	0.026	0.54 [0.21, 1.37]
5	GAD	T0–3	−0.80	0.37	0.031	0.016	0.45 [0.22, 0.93]
		T3–4	0.19	0.41	0.643	0.048	1.21 [0.54, 2.73]
		T4–5	−0.02	0.41	0.959	0.050	0.98 [0.44, 2.19]
6	Specific phobia	T0–3	−0.50	0.47	0.292	0.032	0.61 [0.24, 1.53]
		T3–4	−0.64	0.58	0.273	0.030	0.53 [0.17, 1.65]
		T4–5	0.83	0.58	0.149	0.020	2.30 [0.74, 7.14]
6	Alcohol use disorder	T0–3	−3.02	1.08	0.005	0.008	0.05 [0.01, 0.41]
		T3–4	0.88	1.03	0.392	0.034	2.42 [0.32, 18.27]
		T4–5	−2.01	1.24	0.106	0.020	0.13 [0.01, 1.53]
7	Drug use related disorder	T0–3	−2.38	1.14	0.036	0.018	0.09 [0.01, 0.86]
		T3–4	1.64	1.14	0.149	0.022	5.15 [0.56, 47.74]
		T4–5	−0.53	1.03	0.606	0.046	0.59 [0.08, 4.42]
8	Avoidant personality disorder	T0–T4	−1.30	0.48	0.007	0.008*	0.27 [0.11, 0.70]
9	Paranoid personality disorder	T0–T4	−1.00	0.45	0.025	0.014	0.37 [0.15, 0.88]
10	Obsessive-compulsive personality disorder	T0–T4	−0.53	0.78	0.499	0.040	0.59 [0.13, 2.73]
11	Borderline personality disorder	T0–T4	−0.33	0.38	0.382	0.032	0.72 [0.35, 1.50]

*= Significant effect, Benjamini Hochberg corrected significance level q < .009, OR= odds ratio, 95% CI = 95% confidence interval, MDD = major depressive disorder, GAD.

## Discussion

In the present study, we investigated the effects of prolonged exposure (PE) on comorbid psychological disorders in patients with childhood abuse-related posttraumatic stress disorder (CA-PTSD). Consistent with our hypotheses, PE resulted in a significant decrease in the number of patients who fulfilled diagnostic criteria of depression, anxiety, substance use and personality disorders. We did not find an additional decrease during the follow-up period for most disorders except for depression, nor did we find an increase in this period indicating that any improvements at post-treatment were sustained but no additional improvement occurred. We did not identify changes in the presence of the diagnosis of OCD, psychotic disorders and eating disorders from pre- to post-treatment nor did we identify changes during the follow-up period. We conclude that PE led to substantial and sustained reductions in the diagnosis of the most comorbid disorders.

The effect of PE on the loss of diagnosis with regard to depressive, anxiety, substance use and personality disorders is in line with our expectations. Interestingly, we found that most loss of diagnosis of comorbid disorders occurred from baseline to post-treatment rather than during the follow-up period. Hence, the treatment effect on these comorbid symptoms seems to be relatively fast in line with the effect on PTSD symptoms and loss of PTSD diagnosis (Oprel et al., 2021). There are several potential mechanisms which might explain the treatment effect on comorbid psychopathology. Firstly, PE is a specific type of cognitive behavioral therapy and might directly target symptoms from disorders other than PTSD through shared elements of the treatment. Cognitive behavioral therapy is also the guideline treatment for other disorders such as depression and anxiety, and all types of cognitive behavioral therapy include elements of exposure, either via in vivo assignments or behavioral experiments. Secondly, alleviations in PTSD symptoms might in turn affect other psychopathology since people might experience relief, control over their life and a sense of accomplishment. Previous studies have shown that a reduction of PTSD symptoms resulted in a subsequent reduction in depressive symptomatology (Aderka et al., 2013; McLean et al., 2017). According to the anhedonia model of depression, anhedonia plays a causal role in the development of depression (Loas, 1996). During PE, reductions in trauma-related anxiety and avoidance may lead to an increase in feelings of joy and pleasure, and, as such, reduce anhedonia and, subsequently, depression. Likewise, the helplessness-hopelessness theory stresses that experiencing feelings of helplessness, which are primarily related to (trauma-related) anxiety, can lead to feelings of hopelessness, feelings that are primarily related to depression (Alloy et al., 1990). During PE feelings of helplessness might reduce, which can lead to a subsequent reduction in feelings of hopelessness. In line with this theory, hopelessness has been found to mediate the treatment outcomes of PE for PTSD (Gallagher & Resick, 2012). Thirdly, PTSD symptoms might have been a predisposing and maintaining factor for comorbid conditions and therefore reducing PTSD symptoms might also reduce comorbid symptomatology (Lockwood & Forbes, 2014).

In contrast to our expectations, we did not find a loss of diagnosis in comorbid OCD, psychotic disorders and eating disorders in our sample. Note that the numbers of patients with eating disorders and psychotic disorders were very small in the current study (both below 20), probably explaining the null findings. The findings might also be explained by the treatment length in the current study (only 4–16 weeks), which is shorter than the average treatment length for psychotic and eating disorders (Lincoln et al., 2016; Murphy et al., 2010). Previous studies found that trauma-focused treatment (TFT) led to improvements in psychotic symptoms (de Bont et al., 2016; van den Berg et al., 2018). However, as these studies did not assess clinical diagnoses, results cannot be directly compared.

In the exploratory subgroup analyses, we found a decrease in MDD, agoraphobia, panic disorder, alcohol use disorder and avoidant personality disorder, but we did not find significant changes in the presence of social anxiety disorder, drug use disorder, paranoid personality disorder, obsessive-compulsive personality disorder and borderline personality disorder diagnoses. These results have to be interpreted with caution since the base rate of specific diagnoses was relatively low. A recent study in which all participants were diagnosed with PTSD and a comorbid borderline personality disorder at baseline found that TFT resulted in changes in comorbid diagnostic status of borderline personality disorders at 1-year follow-up (Kolthof et al., 2022). A recent systematic review of the treatment of PTSD and comorbid borderline personality disorder concluded that TFT alone may be a promising treatment for treating both PTSD and borderline personality disorder, but evidence is still very limited (Zeifman et al., 2021). Hence, more studies are needed to draw conclusions about the use of TFT for both PTSD and borderline personality disorder.

### Strengths, limitations and future research

The present study was among the first to investigate the effects of PE on clinician-assessed diagnostic status of comorbid disorders in patients with PTSD in a large sample measured repeatedly over time during and after treatment. Most previous studies used self-reports of comorbid symptom levels. Our study extends the existing literature by using clinical interviews to assess comorbid disorder diagnostic status, and therefore results are more reliable and more likely to reflect an effect of PE beyond PTSD (Engelhard et al., 2007; Franklin & Raines, 2019). Despite these strengths, some limitations need to be acknowledged. Firstly, our study was not powered to detect changes in comorbid disorders, which are relatively rare in patients with PTSD. We performed a power analysis with the assumption that disorders would be present in 25% of the patients at baseline, but some disorders were less common, such as eating disorders and psychotic disorders. The non-significance of the results for these disorders might be due to limited power. Future studies might oversample patients with PTSD and a specific comorbid disorder, for instance as has been done for psychotic disorders (de Bont et al., 2016) and borderline personality disorder (Kolthof et al., 2022), to better understand whether TFT results in improvements in specific comorbid disorders. Secondly, the follow-up period might have been too short to detect change in most personality disorders. Future studies with a longer follow-up period (>12 months) might elucidate the effects of TFT on personality disorders in the long term. Thirdly, patients were allowed to start subsequent treatment for PTSD or comorbid conditions between the 6-month and 12-month follow-up period. The majority of the participants (62% without statistical differences between conditions) received at least one session with a psychologist during this period. On average, these participants received 11.8 sessions (median = 8) between the 6- and 12-month follow-up. The outcomes at the 12-months follow-up therefore cannot solely be attributed to the PE provided in the current study. Lastly, a limitation of the current study is the missing data in follow-up measurements. About one-third of the participants (*n* = 53) did not complete the post-treatment assessment. See Oprel et al. (2021) for a detailed description of the reasons for dropout. All analyses were based on an intention-to-treat sample using maximum likelihood estimation to deal with missing data. In the current sample, comorbidity was the rule rather than the exception. The finding that treatment focused on reducing PTSD symptoms also affects many comorbid diagnoses emphasizes the strong connection between PTSD and comorbid disorders. Future studies could elucidate whether the therapy effect on comorbid disorders is specific for PTSD treatment—implying that PTSD should be the first focus of therapy when PTSD and comorbid diagnoses are present—or whether treatment primary focusing on another diagnoses, such as depression or anxiety, also leads to a reduction in PTSD diagnoses.

### Clinical implications and conclusions

The findings of the current study demonstrate that exposure-based treatment for PTSD, i.e. PE, has a significant beneficial effect on comorbid depressive disorders, anxiety disorders, SUDs and personality disorders in patients with CA-PTSD. This is relevant information both for patients and clinicians since it implies that the effect of exposure-based treatment for PTSD is not limited to PTSD itself but also reduces the presence of comorbid disorders. No treatment effects were found for comorbid OCD, eating disorders and psychotic disorders, possibly due to lack of statistical power in the current study. Despite the positive effects of PE on of comorbid disorders, in addition to the reduction of PTSD, there is room for further improvements. For some patients, psychological problems, PTSD and/or comorbid disorders, were still present after treatment. As such, it is recommended to discuss follow-up treatment options with patients who still meet diagnostic criteria for comorbid disorders after exposure-based treatment for PTSD.

## Data Availability

Proposals for the use of data and requests for access should be directed to vanderdoes@fsw.leidenuniv.nl.
